# Tongue Function: An Underrecognized Component in the Treatment of Obstructive Sleep Apnea with Mandibular Repositioning Appliance

**DOI:** 10.1155/2018/2157974

**Published:** 2018-11-06

**Authors:** Wei Wang, Changping Di, Skaff Mona, Lin Wang, Mark Hans

**Affiliations:** ^1^Jiangsu Key Laboratory of Oral Diseases, Nanjing Medical University, Nanjing, China; ^2^Department of Orthodontic, Affiliated Hospital of Stomatology, Nanjing Medical University, Nanjing, China; ^3^Nanjing Jinling Dental Hospital, Nanjing, China; ^4^Department of Orthodontics, Case Western Reserve University, Cleveland, USA

## Abstract

Obstructive sleep apnea (OSA) is a common but still underrecognized disorder. A mandibular repositioning appliance (MRA) is used to treat OSA by advancing the mandible and thereby reducing the collapsibility of the upper airway. It has been found that an MRA increases the volume of the upper airway, especially the velopharyngeal area, in OSA patients. We hypothesize that this increase in the velopharyngeal volume is associated with an anterior displacement of the tongue, but likely not with a stretching of the soft tissue connecting the soft palate, lateral pharynx, palatopharyngeal arch, and mandible. Since the function and structure of the genioglossus and hypoglossal nerve are always abnormal in patients with OSA, the tongue does not always move simultaneously with the mandible when an MRA is being used. Oropharyngeal exercises, especially tongue exercises, can improve the quality of life of OSA patients, including reduction of daytime sleepiness and snoring, better quality of sleep, and partial decrease in the AHI. Further, in animal models, tongue exercise is also found to be effective in tongue function recovery and in the remodeling of the hypoglossal nucleus. We suggest that a combination of tongue exercises along with MRA is a promising approach for patients who do not respond to an MRA alone.

## 1. Introduction

Obstructive sleep apnea (OSA) is a common but still underrecognized disorder affecting an estimated 2% of middle-aged women and 4% of middle-aged men [1]. In addition, as a precursor to OSA, loud snoring affects almost 40–60% of all adults [2].

During OSA, the upper airway collapse most often results from a combination of anatomic factors plus neuromuscular compensation, a combination that is insufficient during sleep to maintain airway potency [3], but the mechanism itself is still not clear.

Continuous positive airway pressure (CPAP) has proved to be an effective treatment for OSA patients in several studies [4–6]. Patient compliance and long-term acceptance, however, are relatively low since CPAP is an uncomfortable and inconvenient burden [7, 8].

Oral appliance therapy has also been used for OSA patients since it has fewer side effects, provides better tolerance, and offers greater personal satisfaction [9]. The most common type of oral appliance is the mandibular repositioning appliance (MRA), which is usually used to advance the mandible and thereby reduce the collapsibility of the upper airway. The MRA has been effective in mild, moderate, and even severe OSA patients. The mechanism of action, however, is less predictable than that of CPAP. On average, a complete response (resolution of OSA or an AHI <5 events/h) occurred in around 48% of patients after MRA treatment [10].

In most test groups with OSA, the oral pharyngeal region is smaller than that of the control group, and so closure is more common. This fact has been demonstrated in many studies by using nasal pharyngoscopy (NP), computer tomography (CT), magnetic resonance imaging (MRI), or pharyngeal pressure monitoring [11–14].

The tongue is known to be the most important pharyngeal dilator muscle [15], which is unique and moves freely, unlike other muscles, which are anchored to the bone at one end only. It has been found that an MRA can enlarge the upper airway by moving the entire tongue forward [16].

Therefore, this review discusses the key effects on the tongue when an MRA is used to treat OSA.

## 2. Anatomic Structure of the Tongue

The tongue is formed by extrinsic and intrinsic muscles. Generally, the extrinsic muscles—including the genioglossus, the hyoglossus, the styloglossus, and the palatoglossus—tend to move the position of the whole tongue, while the intrinsic muscles change its shape. The genioglossus arises from the mental symphysis and enters the dorsum of the tongue; the primary function of it is protruding and depressing the tongue. The hyoglossus arises from the hyoid bone and enters the side of the tongue; the primary function of it is depressing and retracting the tongue. The styloglossus originates at the styloid process of the temporal bone and enters the side of the tongue; the main function of it is retracting and elevating the tongue. The palatoglossus arises from the palatine aponeurosis and enters broadly across the tongue; the main function of it is elevating the posterior aspect of the tongue. The genioglossus, the hyoglossus, and the styloglossus are innervated by the hypoglossal nerve, while the palatoglossus is innervated via the vagus nerve.

Since the tongue, mandible, and hyoid are connected and thus they form a complex biomechanical system, the functional connections among these three anatomical structures need to be taken into account [17].

**Figure 1:**
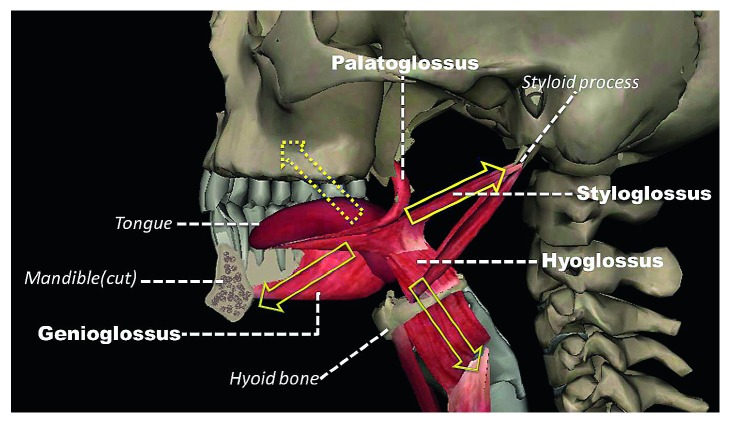
The tongue is hanging between the mandibular, hyoid, and maxillary complex. There is no muscle in this direction (yellow dotted arrow). It is easy for the tongue to move downward and backward (yellow arrow).

As shown in Figure 1, the tongue is suspended within the bony framework that includes the mandible, the hyoid bone, and the cranial base. It is evident that moving the tongue forward and upward is difficult since there is almost no muscle pull in these directions.

## 3. An MRA Increases the Upper Airway Volume by Changing the Position of the Tongue

Many studies have reported that an MRA increases the upper airway volume in patients with OSA [18–22] or patients with mandibular retrognathia [23–25].

It has been suggested that the mechanism of action of an MRA serves to increase the volume of the upper airway in OSA patients [26]. Thus, this increase in the volume is associated with an anterior displacement of the tongue, since the genioglossus, as one of the tongue's major muscles, is attached to the lingual surface of the anterior mandibular arch [27].

Tongue retaining devices (TRDs), another oral appliance used for OSA, improve upper airway potency by anteriorly displacing the tongue with suction forces while patients sleep. A study using MRI has found that TRDs have a greater effect on upper airway size, especially the velopharyngeal volume, than an MRA. One of the reasons for this difference is that the forward tongue movement causes a greater increase in the oropharyngeal and velopharyngeal cross section area than does mandibular protrusion alone [20].

In normal function, the tongue should be positioned upward against the palate at rest. Nasal obstruction results in a constricted and V-shaped maxillary dental arch and a lower tongue posture. After rapid maxillary expansion, the tongue is able to move upward, and the upper airway volume becomes enlarged [28].

Thus, mandibular advancement causes the tongue to move forward or upward, which, though beneficial for improving the upper airway, is unpredictable. Brown et al. used the spatial modulation of magnetization to dynamically image the movement of the tissues surrounding the airway during mandibular advancement [16]. They found that an MRA can enlarge the upper airway by moving the entire tongue forward [16]. Whole tongue movement forward, however, happened in only 33% of the subjects using mandibular advancement [16]. Almost 40% of the subjects showed just a minimal anterior displacement of the posterior part of the tongue [16]. Sutherland et al. used MRI to establish that the tongue moved forward only 0.06 ± 0.04 cm but downward 0.11 ± 0.06 cm after MRA treatment [20]. The inconsistent movement of the tongue may be the reason why the oropharynx volume did not significantly change in these studies [20, 25].

As a result, changing the tongue position, especially the posterior part, forward and upward is beneficial for increasing the upper airway volume when using an MRA to treat OSA patients.

## 4. An MRA Increases the Velopharynx Volume by Moving the Tongue Away from Soft Palate: A Hypothesis

An MRA has been found to improve the velopharynx airway patency in anesthetized, completely paralyzed [29], and awake [30] patients with OSA. It has been suggested that the mechanism of action of an MRA serves to increase the volume of the upper airway, predominantly as a result of an increase in the velopharynx but not the oropharynx volume [20, 26, 31].

This increase is likely a consequence of stretching the soft tissue connecting the soft palate, lateral pharynx, tongue, and palatopharyngeal arches [29, 32]. This mechanism, however, is still not clear.

**Figure 2:**
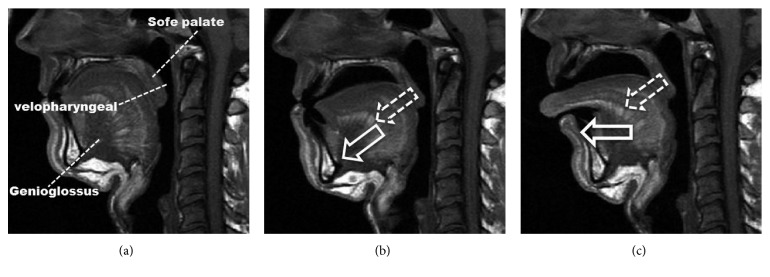
(a) Patient without treatment. (b) Patient with MRA treatment. (c) Patient with TRD treatment. The more the tongue moves forward and downward (white arrow), the more the soft palate moves anterior and superior (white dotted arrow).

Here we propose a hypothesis, as shown in Figure 2(b) [20]. An MRA maintains the mandible in a protruded position. The genioglossus causes the tongue to move forward and downward so that it is held away from the soft palate; therefore, the pressure on the soft palate is released, and in this way, it moves anteriorly and superiorly, and thus produces an increase in the velopharyngeal volume, which may be more apparent if the tongue moves more forward (Figure 2) [20]. The hypothesis may hold true especially for those who have a big tongue or mandibular retrognathia. Although this hypothesis is not proven, the soft palate moves anteriorly and superiorly in coordination with the tongue's anterior movement [20]. In addition, airway closure in obese OSA subjects occurs primarily at the velopharynx [33], which may be the reason for more pressure on the soft palate, since obese OSA patients have an increased tongue volume and deposition of fat at the base of the tongue [34]. Last but not least, the hypothesis might be tested by a study comparing MAD alone with MAD combined with oropharyngeal exercises.

## 5. Why Does the Tongue Not Always Move Simultaneously with Mandibular Movement in OSA Patients Using an MRA?

The patency of the airway, which depends on the tonic and phasic activation of muscles of the tongue and pharynx, is supplied by the pontomedullary cranial nerves [35]. Damage to the hypoglossal nerve at the level of the medulla would contribute to severe obstructive sleep apnea [36], which is relevant in this discussion because hypoglossal nerve conduction has been found in OSA patients [37].

The genioglossus, which is innervated by the hypoglossal nerve, is considered the main tongue protruder and main pharyngeal dilator [38]. The function and structure of the genioglossus and hypoglossal nerve, however, are always abnormal in patients with OSA. Compared with normal subjects, OSA subjects exhibit chronic neuromuscular injury of the genioglossus, especially the posterior of the genioglossus [39]. Carrera et al. found that the genioglossus muscle in patients with OSA reveals greater fatigability [40]. Another study using electromyography showed that most OSA patients have significantly impaired hypoglossal nerve conduction in the form of delayed distal latency and low motor amplitude [41].

On the contrary, other studies have reported that the type II fibers, which are less resistant to fatigue, are increased in individuals with sleep apnea [40, 42]. As a result, the tongue is more prone to fatigue in patients with OSA and is less stiff than in healthy subjects; this fatigue occurs in the muscle fiber direction, which probably contributes to increased collapsibility of the airway [43].

It has been unclear until now whether the hypoglossal axonal damage is homogeneous or not. Cranial hypoglossal axonal motor neuropathy has been found in patients with OSA [37]. This discovery has been substantiated by another study with similar subjects who have not only a sensory axonal and motor axonal but also peripheral polyneuropathy [44].

In summary, the hypoglossal axonal damage in patients with OSA may lead to uncoordinated tongue movement with mandibular advancement.

## 6. How to Move the Tongue Forward in Coordination with Mandibular Advancement in OSA Patients?

The key for moving the tongue forward with mandibular advancement in OSA patients is to correct the hypoglossal axonal damage. No evidence shows, however, that the hypoglossal axonal damage can be corrected clinically. Hypoglossal nerve stimulation is an alternative therapy that can directly open the hypopharyngeal airway with tongue protrusion. Nevertheless, complications such as infection, permanent hypoglossal nerve damage, and postoperative discomfort and limitations such as narrow selection criteria and the high cost of the hypoglossal nerve stimulation implant hinder wide application [45].

Oropharyngeal exercises are a set of isometric and isotonic exercises involving the tongue, soft palate, and lateral pharyngeal wall; the purpose of the exercises is to change orofacial muscular and functional patterns including those of suction, swallowing, chewing, breathing, and speaking [46].

Applied to OSA subjects, these exercises are believed to strengthen the nasopharynx and oropharynx musculature and thus contribute to the reduction in the collapse of the airway during sleep [46–50].

A randomized trial (RCT) showed that 3 months of oropharyngeal exercises lead to significant reductions—the frequency of snoring by 36% and the total power of snoring by 59%—in patients with primary snoring and mild to moderate OSA [47].

Another RCT reported a decrease of approximately 40% in the Apnea-Hypopnea Index (AHI), a reduction in cervical circumference and in snoring intensity and frequency, and a decrease in daytime sleepiness. The RCT made clear that better sleep quality results from oropharyngeal exercises performed by patients with moderate OSA [46].

Nevertheless, the exact mechanisms of the oropharyngeal exercises are still not clear, but the exercises are effective in many patients with snoring and mild, moderate, or even severe OSA [49–53]. Furthermore, these exercises can promote remodeling of the upper airway if they are performed by the patient with constant and reliable frequency (two or three times a day); lack of such frequency may limit the clinical applicability [54].

Physiologically, the pharynx is a highly collapsible area because it lacks rigid supporting structures. But the dilating pharynx muscles, especially the genioglossus, prevent the tendency of the pharynx to collapse. As a result, tongue practice should be the priority of oral exercises because of its direct effect on the hypoglossal-genioglossus axis. In animal models, tongue practice has been found to be effective in tongue function recovery [55] and remodeling [56–58] the hypoglossal nucleus.

In addition, a pilot study has found that AHI decreases from 20.9 ± 5.3 to 16.1 ± 5.1 events/h after only one-week tongue training in severe OSA subjects [59].

## 7. How to Do Tongue Exercises?

Generally speaking, tongue exercises consist of moving the tongue in different directions with or without sticking the tongue out, sucking the tongue against the palate, pressing the tongue against bony and soft tissue structures within the oral cavity, and doing other tongue movements with or without resistance [60].

Studies indicate that the tongue-pressure-on-the-palate exercises are effective in the rehabilitation of lingual strength [61, 62] and promote substantial activation of suprahyoid musculature [62]. As a result, the exercises include moving the tongue forward and upward, which can be the most effective way for a referral exercise to benefit every muscle connected to the whole system: the tongue, the mandible, and the hyoid (Figure 1).

## 8. Conclusion

Oropharyngeal exercises can improve the quality of life of OSA patients, including reduction of daytime sleepiness and snoring, better quality of sleep, and partial decrease in the AHI and thereby partial increase in the blood minimum saturation. The exercises, however, cannot lead to a complete recovery for OSA patients [63].

An MRA is a removable, noninvasive, intraoral dental splint used to protrude the mandible in a forward position. On average, a complete response (resolution of OSA or an AHI <5 events/h) occurs in around 48% of patients after MRA treatment [10].

Because using combination therapies to treat OSA is a promising approach [54], oropharyngeal exercises may be an effective complementary way for patients who do not respond to an MRA.

In addition, thanks to the genioglossus, it is easy for the tongue to move downward with an MRA. Here we present a hypothesis that MRA may be beneficial for releasing the pressure on the soft palate and increasing the velopharyngeal volume by moving the tongue forward and downward. However, the hypothesis should be corroborated by a clinical study.

On the contrary, since mandibular advancement causing the tongue to move forward or upward is unpredictable, tongue exercises may be a beneficial way to recover tongue function or to remodel the hypoglossal nucleus; therefore, the most feasible way to move the tongue forward and upward is through the use of an MRA.
